# The Increased Risk of Thyroid Cancer-Specific Mortality With Tumor Size in Stage IVB Patients

**DOI:** 10.3389/fonc.2020.560203

**Published:** 2020-11-05

**Authors:** Junyi Zhang, Xiaoyun Cheng, Bin Su, Xingchun Wang, Lu Wang, Muthukumaran Jayachandran, Xiaoting Sun, Le Bu, Yueye Huang, Shen Qu

**Affiliations:** ^1^Department of Endocrinology and Metabolism, Shanghai Tenth People's Hospital, Tongji University School of Medicine, Shanghai, China; ^2^Nanjing Medical University, Nanjing, China; ^3^Department of Endocrinology and Metabolism, The Shanghai Tenth People's Hospital Chongming Branch, Tongji University School of Medicine, Shanghai, China; ^4^Shanghai Center of Thyroid Disease, Shanghai, China; ^5^Shanghai Tenth People's Hospital, Tongji University School of Medicine, Shanghai, China

**Keywords:** thyroid cancer, stage IVB, tumor size, SEER, cancer-specific mortality

## Abstract

**Purpose:** To investigate the risk-stratifying utility of tumor size and a threshold for further stratification on cancer-specific mortality of thyroid cancer (TC) patients in stage IVB.

**Methods:** One thousand three hundred and forty-five patients (620 males and 725 females) with initial distant metastasis over 55 years between 2004 and 2016 from Surveillance, Epidemiology, and End Results databases were investigated, with a median follow-up time of 23 months [interquartile range (IQR), 5–56 months] and a median age of 70 years (IQR, 63–77 years). TC-specific mortality rates were calculated under different classifications. Cox regressions were used to calculate hazard ratios (HRs) and Kaplan-Meier Analyses were conducted to investigate TC-specific survivals.

**Results:** In the whole cohort, patients with tumors >4 cm had the highest TC-specific mortality (67.9%, 330/486), followed by tumor size >1 cm but ≤ 4 cm (43.08%, 190/441), and tumor size ≤ 1 cm (32.69%, 34/104). Kaplan-Meier curves showed the increased tumor size was associated with a statistically significant decrease in TC-specific survival (*P* < 0.001). Papillary thyroid cancer (PTC) patients with tumors >4 cm had significantly higher hazard ratios (HRs) of 2.84 (1.72–4.70) and 3.11 (1.84–5.26) after adjusting age, gender, race, and radiation treatment, compared with patients with tumors ≤ 1 cm (*P* < 0.001). The TC-specific mortalities and survivals were further investigated among more detailed subgroups divided by different tumor size, and a threshold of 3 cm could be observed (*P* < 0.005) for risk stratification.

**Conclusions:** Mortality risk increased with tumor size in PTC patients in stage IVB. Our findings demonstrated the possibility of further stratification in IVB stage in current TNM staging system. Patients with tumor size over 3 cm had an excessively high risk of PTC-specific mortality, which may justify the necessity of more aggressive treatment for them.

## Introduction

Thyroid cancer (TC) is the most common endocrine malignancy increasing rapidly over the past decades and associated with a favorable prognosis (1–5). Differentiated thyroid cancers (DTC), which consist of papillary thyroid cancer (PTC), and follicular thyroid cancer (FTC), account for ~>90% of TC (6, 7). However, patients with initial distant metastasis still suffer a high risk of mortality (8–10). About 10–15% of patients with TC are at an advanced stage (11). Advanced stage TC is associated with a 5-years survival rate of <50%, which was apparently lower than the survival rate for patients without an aggressive form of TC (12).

The American Joint Committee on Cancer (AJCC) Staging Manual (13) is the most widely applied evidence-based staging system for the assessment of TC. Initially, the definition of stage IVB DTC, defined as patients aged 45 years and older with T4b, any N, and M0, first appeared in the AJCC Staging Manual 6th edition with the age limitation of 45 years and older and remained unchanged in the 7th edition. But it was later changed into any T, any N, and M1 with the age limitation changed into 55 years and older in the 8th edition (14). DTC patients in stage IVB diagnosed with distant metastases were extrapolated to have excess mortality risk, which made tumor size less important. However, even with the same TNM stage, the prognosis for patients still remains controversial (15–17). Recent studies (18, 19) have been conducted to determine the influence of tumor size on the cancer-specific survival of patients in stage IVB. It has been illustrated that the larger tumor size may be associated with a higher risk of cancer-specific mortality in stage IVB TC patients. Therefore, further stratification by tumor size is required for these patients in stage IVB.

The ultimate aim of this retrospective study is to determine the prognostic value of tumor size in thyroid cancer patients in stage IVB and thyroid cancer variants, and how tumor size could guide the treatment strategies.

## Materials and Methods

### Patients and Clinicopathological Data

A retrospective study was conducted to investigate the association between tumor size and the prognosis among patients in stage IVB. We retrieved data from patients aged over 55 years with distant metastasis who met the criteria for stage IVB identified by the AJCC Staging Manual 8th edition (13). All data were obtained from the Surveillance, Epidemiology, and End Results (SEER) database (https://seer.cancer.gov/) using the SEER^*^Stat 8.3.5 software (20), which is a United States (US) population-based cancer registry that began in 1,973 and includes ~34.6% of the US population. All cases and its variants were classified using the 3rd edition of the International Classification of Diseases for Oncology. One thousand three hundred and forty-five cases with complete clinical characteristics were investigated. Three hundred and fourteen patients with two or more primary tumors were excluded.

Patients were divided into three groups based on tumor size as following: tumors ≤ 1 cm, tumors >1 cm but ≤ 4 cm, and tumors >4 cm group. They were analyzed in each different lymph node (N) categories. The definition of N categories were classified by the 6th AJCC Staging Manual (21) with no metastatic nodes (N0), nodes not assessed at surgery (Nx), metastases to level VI [pretracheal, paratracheal, and prelaryngeal/Delphian lymph nodes] (N1a), metastasis to unilateral, bilateral, or contralateral cervical or superior mediastinal lymph node metastases (N1b), and metastasis to regional lymph nodes but not otherwise specified (N1NOS), and the definition of M1 was patients with distant metastasis.

### Statistical Analyses

Kaplan-Meier analyses were conducted to assess the survival of different tumor size groups and its variants along with log-rank tests. The effect of tumor size on TC-specific mortality was evaluated using Cox regression, and the hazard ratios (HRs) and 95% confidence intervals (CIs) were calculated. A two-tailed *p* < 0.05 was considered statistically significant. All data were analyzed using the Statistical Package for Social Science version 25.0 (SPSS, Inc., New York, NY, USA).

## Results

### Demographic and Clinical Characteristics

The clinical characteristics of all TC patients in stage IVB were shown in Table 1, including 1,345 cases with a median age of 70 years (interquartile range, 63–77 years). The majority of cases were classified into the tumors >4 cm group (46.17%, 621/1,345), followed by tumors >1 cm but ≤ 4 cm group (42.75%, 575/1,345) and tumors ≤ 1 cm group (11.08%, 149/1,345). The rates for PTC, FTC, and anaplastic thyroid cancer (ATC) were 56.73% (763/1,345), 19.26% (259/1,345), and 8.55% (115/1,345), respectively. In all N categories, the majority of cases of PTC, FTC, ATC, and other variants involved tumors >4 cm [incidence rates of 41.38% (257/621), 22.06% (137/621), 15.14% (94/621), and 21.42% (133/621), respectively]. The overall mortality in all TC cases was 53.73% (554/1,031). The patients with tumors >4 cm had the highest mortality rate of 67.90% (330/486), followed by tumors >1 cm but ≤ 4 cm (43.08%, 190/441) and ≤ 1 cm (32.69%, 34/104) group. Similar results were obtained in PTC and FTC (Table 1). A total of 39.63% (533/1,345) patients received radioactive iodine therapy or radiation beam plus isotopes or implants, which refer to patients with records in SEER dataset named “Combination of beam with implants or isotopes” and “Radioisotopes (1988+).” A total of 24.91% (335/1,345) patients received therapy of radiation beam or radioactive implants. The rates of patients with tumors >4 cm were 33.68% (257/763), 52.90% (137/259), and 81.74% (94/115) for PTC, FTC, and ATC, respectively. Similar results were obtained in each N category (Supplementary Table 1).

**Table 1:** The clinical characteristics of thyroid cancer with increased tumor size (SEER database years of 2004−2016).

**Characteristics**		**Overall**		**≤1 cm**		**>1 cm but** **≤4 cm**		**>4 cm**	
**Number**		**1,345**		**149**		**575**		**621**	
		***N***	**%**	***N***	**%**	***N***	**%**	***N***	**%**
Age (y)	Median (IQR)	70 (63–77)		69 (62–77)		70 (63–77)		70 (63–78)	
Gender	Male	620	46.10	62	41.61	255	44.35	303	48.79
	Female	725	53.90	87	58.39	320	55.65	318	51.21
Race	White	993	73.83	111	74.50	421	73.22	461	74.24
	Black	132	9.81	16	10.74	46	8.00	70	11.27
	Others	217	16.13	20	13.42	108	18.78	89	14.33
	Unknown	3	0.22	2	1.34	0	0.00	1	0.16
Lymph node stage	N0	535	39.78	82	55.03	255	44.35	198	31.88
	N1a	156	11.60	13	8.72	72	12.52	71	11.43
	N1b	419	31.15	26	17.45	168	29.22	225	36.23
	N1NOS	95	7.06	12	8.05	33	5.74	50	8.05
	NX	140	10.41	16	10.74	47	8.17	77	12.40
Extrathyroidal extension	No	536	39.85	121	81.21	261	45.39	154	24.80
	Yes	735	54.65	27	18.12	276	48.00	432	69.57
	Unknown	74	5.50	1	0.67	38	6.61	35	5.64
Thyroid cancer specific mortality^a^	alive	477	46.27	70	67.31	251	56.92	156	32.10
	death	554	53.73	34	32.69	190	43.08	330	67.90
Histology subtype	PTC	763	56.73	101	67.79	405	70.43	257	41.38
	FTC	259	19.26	31	20.81	91	15.83	137	22.06
	ATC	115	8.55	3	2.01	18	3.13	94	15.14
	Other	208	15.46	14	9.40	61	10.61	133	21.42
Thyroid cancer specific mortality^a^	TC	554	53.73	34	32.69	190	43.08	330	67.90
	PTC	234	22.70	18	17.31	110	24.94	106	21.81
	FTC	91	8.83	6	5.77	26	5.90	59	12.14
	ATC	85	8.24	1	0.96	16	3.63	68	13.99
	Other	144	13.97	9	8.65	38	8.62	97	19.96
Radiation therapy									
Radiation Beam or Radioactive implants		335	24.91	32	21.48	109	18.96	194	31.24
Radioisotopes or Radiation beam plus isotopes or implants		533	39.63	70	46.98	285	49.57	178	28.66
None or refused		445	33.09	42	28.19	171	29.74	232	37.36
Unknown		32	2.38	5	3.36	10	1.74	17	2.74

*IQR, interquartile range; PTC, papillary thyroid cancer; FTC, follicular thyroid cancer; ATC, anaplastic thyroid cancer; Other, other variants of thyroid cancer. According to the American Joint Committee on Cancer (AJCC) Staging Manual 6th Edition, lymph node category was classified into 5 groups as follows: no metastatic nodes (N0); nodes not assessed at surgery (Nx); metastases to level VI [pretracheal, paratracheal, and prelaryngeal/Delphian lymph nodes] (N1a); metastasis to unilateral, bilateral, or contralateral cervical or superior mediastinal node metastases (N1b); and metastasis to regional lymph nodes but not otherwise specified (N1NOS)*.

a *314 patients with the status of cancer-specified death remaining unknown were excluded, which included 45 patients with tumors ≤ 1 cm, 134 patients with tumors >1 cm but ≤ 4 cm, and 135 patients >4 cm*.

### The Relationship Between TC-Specific Mortality and Increased Tumor Size

The overall TC-specific mortality rate was 53.7% (554/1,031), and was 32.69% (34/104), 43.08% (190/441), 67.90% (330/486) for patients with tumors ≤ 1 cm, >1 cm but ≤ 4 cm, and > 4 cm. Compared with tumors ≤ 1 cm group, the crude HRs were 1.35 (0.94–1.95, *P* = 0.104) and 3.08 (2.16–4.38, *P* < 0.001) for tumors >1 cm but ≤ 4 cm and tumors >4 cm group. After adjustments for age, gender, race, and radiation treatment, the HRs were 1.51 (1.04–2.20, *P* = 0.029) and 3.11 (2.17–4.47, *P* < 0.001) for tumors >1 cm but ≤ 4 cm and >4 cm group. In order to eliminate the effect of the lymph node metastases, we further investigated the risk of TC-specific mortality in each N category. In N0 category, the TC-specific mortalities were 28.07% (16/57), 39.50% (79/200), and 57.14% (88/154) for tumor ≤ 1 cm, >1 cm but ≤ 4 cm, and >4 cm group. Compared with tumors ≤ 1 cm group, tumors >1 cm but ≤ 4 cm group did not have any prognostic value in TC-specific death. However, tumors >4 cm group had significantly higher HRs for TC-specific mortality compared with tumors ≤ 1 cm [crude HR 2.38 (1.40–4.06), *P* = 0.001; adjusted HR 2.97 (1.72–5.12), *P* < 0.001]. Similar results were obtained in the N1 and Nx category.

The overall PTC-specific mortality was 41.71% (234/561), and was 26.09% (18/69), 36.42% (110/302), 55.79% (106/190) for tumors ≤ 1 cm, tumors >1 cm but ≤ 4 cm, and tumors >4 cm, respectively. The crude HR of tumors >4 cm group was 2.84 (1.72–4.70, *P* < 0.001) and the adjusted HR was 3.11 (1.84–5.26, *P* < 0.001), compared with tumors ≤ 1 cm, and they remained significantly in N0 and N1 category but Nx category due to the small number size (Table 2). The overall FTC-specific mortality was 43.96% (91/207), and was 26.09% (6/23), 36.11% (26/72), 52.68% (59/112) for tumors ≤ 1 cm, >1 cm but ≤ 4 cm, and >4 cm group. The crude HR of tumors >3 cm group was 1.82 (1.13–2.92, *P* = 0.014) and the adjusted HR was 1.69 (1.02–2.79, *P* = 0.040) compared with tumors ≤ 1 cm group (Supplementary Table 2).

**Table 2:** The Association between tumor size and thyroid cancer specific mortality in patients with different *N* category (SEER database years of 2004–2016).

**Variants**		**Mortality**	**Unadjusted**		**Adjusted^a^**	
		***n*****/*****N*** **(%)**	**HR (95%CI)**	***P***	**HR (95%CI)**	***P***
TC		554/1,031 (53.7)				
All N	≤ 1 cm	34/104 (32.69)	Ref.
	>1 cm but ≤ 4 cm	190/441 (43.08)	1.35 (0.94–1.95)	0.104	1.51 (1.04–2.20)	0.029
	>4 cm	330/486 (67.90)	3.08 (2.16–4.38)	<0.001	3.11 (2.17–4.47)	<0.001
N0	≤ 1 cm	16/57 (28.07)	Ref.
	>1 cm but ≤ 4 cm	79/200 (39.50)	1.33 (0.78–2.28)	0.294	1.57 (0.91–2.71)	0.107
	>4 cm	88/154 (57.14)	2.38 (1.40–4.06)	0.001	2.97 (1.72–5.12)	<0.001
N1	≤ 1 cm	13/35 (37.14)	Ref.
	>1 cm but ≤ 4 cm	95/204 (46.57)	1.40 (0.78–2.50)	0.256	1.10 (0.61–1.99)	0.749
	>4 cm	199/277 (71.84)	3.32 (1.89–5.84)	<0.001	2.36 (1.33–4.19)	0.003
Nx	≤ 1 cm	5/12 (41.67)	Ref.
	>1 cm but ≤ 4 cm	16/37 (43.24)	1.14 (0.41–3.15)	0.800	2.19 (0.61–7.78)	0.228
	>4 cm	43/55 (78.18)	3.16 (1.24–8.04)	0.016	3.47 (1.18–10.21)	0.024
PTC		234/561 (41.71)				
All N	≤ 1 cm	18/69 (26.09)	Ref.
	>1 cm but ≤ 4 cm	110/302 (36.42)	1.36 (0.83–2.24)	0.227	1.67 (0.99–2.82)	0.053
	>4 cm	106/190 (55.79)	2.84 (1.72–4.70)	<0.001	3.11 (1.84–5.26)	<0.001
N0	≤ 1 cm	8/35 (22.86)	Ref.
	>1 cm but ≤ 4 cm	36/117 (30.77)	1.16 (0.54–2.50)	0.708	1.41 (0.63–3.16)	0.406
	>4 cm	24/51 (47.06)	2.17 (0.97–4.85)	0.059	3.54 (1.46–8.61)	0.005
N1	≤ 1 cm	8/28 (28.57)	Ref.
	>1 cm but ≤ 4 cm	67/168 (39.88)	1.46 (0.70–3.05)	0.313	1.13 (0.54–2.40)	0.742
	>4 cm	76/131 (58.02)	3.00 (1.44–6.25)	0.003	2.30 (1.09–4.88)	0.029
Nx	≤ 1 cm	2/6 (33.33)	Ref.
	>1 cm but ≤ 4 cm	7/17 (41.18)	1.25 (0.25–6.23)	0.786	3.70 (0.32–42.83)	0.295
	>4 cm	6/8 (75.00)	3.76 (0.74–19.02)	0.110	-	-

*PTC, papillary thyroid cancer; FTC, follicular thyroid cancer*;

a *Adjusted for age, gender, race, and radiation treatment*.

### Comparison of PTC-Specific Mortality in Patients With Different Tumor Sizes

The PTC-specific mortality rates were 26.09% (18/69), 30.56% (33/108), 36.04% (40/111), 44.58% (37/83), 47.66 % (183/384), and 52.38% (143/273) for tumors ≤ 1 cm, tumors >1 cm but ≤ 2 cm, tumors >2 cm but ≤ 3 cm, tumors >3 cm but ≤ 4 cm, tumors >2 cm, and tumors >3 cm group, respectively. Apparently, PTC-specific mortality had an increasing tendency with increased tumor size, and this trend was also found when N0 and N1 categories were analyzed alone. Compared with tumors ≤ 1 cm group, tumors >3 cm group had a significant adjusted HR of 2.70 (1.63–4.49, *P* < 0.001), tumors >3 cm but ≤ 4 cm group had an adjusted HR of 2.12 (1.18–3.81, *P* = 0.012). In N0 patients, tumors >3 cm group had significant adjusted HR of 2.71 (1.21–6.06, *P* = 0.015) compared with tumors ≤ 1 cm but this difference lost significance in N1 patients (Table 3).

**Table 3:** Further investigation on the association between tumor size and thyroid cancer specific mortality in papillary thyroid cancer patients with different N category (SEER database years of 2004–2016).

**PTC**	**Mortality**	**Unadjusted**		**Adjusted^a^**	
	***n/N*** **(%)**	**HR (95%CI)**	**P**	**HR (95%CI)**	***P***
All N	234/561 (41.71)				
≤ 1 cm	18/69 (26.09)	Ref.
>1 cm but ≤ 2 cm	33/108 (30.56)	1.10 (0.62–1.95)	0.757	1.58 (0.82–3.05)	0.170
>2 cm but ≤ 3 cm	40/111 (36.04)	1.35 (0.77–2.37)	0.296	1.56 (0.87–2.79)	0.138
>3 cm but ≤ 4 cm	37/83 (44.58)	1.82 (1.04–3.20)	0.037	2.12 (1.18–3.81)	0.012
>2 cm	183/384(47.66)	2.11(1.30–3.43)	0.002	2.24(1.36–3.68)	0.002
>3 cm	143/273 (52.38)	2.48 (1.52–4.06)	<0.001	2.70 (1.63–4.49)	<0.001
N0	68/203 (33.50)	
≤ 1 cm	8/35 (22.86)	Ref.
>1 cm but ≤ 2 cm	14/49 (28.57)	1.10 (0.46–2.64)	0.824	1.61 (0.54–4.79)	0.390
>2 cm but ≤ 3 cm	10/37 (27.03)	1.02 (0.40–2.59)	0.970	0.91 (0.33–2.48)	0.848
>3 cm but ≤ 4 cm	12/31 (41.94)	1.46 (0.59–3.58)	0.414	2.16 (0.77–6.00)	0.142
>2 cm	46/119 (38.66)	1.60 (0.75–3.39)	0.223	1.89 (0.88–4.06)	0.102
>3 cm	36/82 (43.90)	1.87 (0.87–4.04)	0.110	2.71 (1.21–6.06)	0.015
N1	151/327 (46.18)	
≤ 1 cm	8/28 (28.57)	Ref.
>1 cm but ≤ 2 cm	16/53 (30.19)	1.05 (0.45–2.46)	0.914	0.93 (0.37–2.38)	0.886
>2 cm but ≤ 3 cm	28/67 (41.79)	1.54 (0.69–3.40)	0.289	1.19 (0.52–2.71)	0.683
>3 cm but ≤ 4 cm	23/48 (47.92)	1.97 (0.88–4.41)	0.100	1.33 (0.57–3.01)	0.510
>2 cm	127/246 (51.63)	2.30 (1.13–4.71)	0.022	1.84 (0.89–3.79)	0.100
>3 cm	99/179 (55.31)	2.65 (1.29–5.46)	0.008	1.96 (0.94–4.08)	0.071

*PTC, papillary thyroid cancer*.

a *Adjusted for age, gender, race, and radiation treatment*.

### Investigation on the Threshold of Tumor Size Concerning PTC-Specific Mortality in IVB Patients With Different N Categories

The PTC-specific mortality rates were 28.81% (51/177) and 47.66% (183/384) for patients with tumors ≤ 2 cm and >2 cm, which corresponded to a crude HR of 2.04 (1.50–2.79, *P* < 0.001) and an adjusted HR of 1.99 (1.45–2.74, *P* < 0.001), when comparing the latter group with the former. In N0 patients, the risk of PTC-specific mortality did not show any significant difference between patients with tumors ≤ 2 cm and >2 cm. In N1 patients, PTC >2 cm had a significant higher mortality risk, with an adjusted HR of 2.08 (1.33–3.26, *P* = 0.001). The PTC-specific mortality rates were 31.60% (91/288) and 52.38% (143/273) for patients with tumors ≤ 3 cm and >3 cm. PTC patients with tumors >3 cm had a higher adjusted HR of 2.22 (1.69–2.09, *P* < 0.001) and remained significant when each N category was analyzed alone (Table 4). For patients with extrathyroidal extension, the PTC-specific mortality rate of group with tumors >2 cm was 56.15% (146/260) vs. 29.69% (19/64) of group with tumors ≤ 2 cm [HR 2.57 (1.59–4.16), *P* < 0.001; and adjusted HR 2.35 (1.44–3.83), *P* = 0.001], while the PTC-specific mortality rate for tumors ≤ 3 cm group was 41.27% (52/126) vs. 57.07% (113/198) for tumors >3 cm group [HR 1.85 (1.33–2.58), *P* < 0.001; and adjusted HR 1.79 (1.28–2.51), *P* = 0.001]. For patients without extrathyroidal extension, 2 cm showed no prognostic value in risk stratification of PTC-specific mortality. Compared with tumors ≤ 3 cm, tumor size >3 cm group had higher mortality risk with a crude HR of 1.84 (1.12–3.05) (*P* = 0.017) and an adjusted HR of 1.90 (1.12–3.22) (*P* = 0.017) (Table 5). For FTC cases, a threshold of 3 cm could also be observed (Supplementary Table 2).

**Table 4:** The threshold of tumor size concerning thyroid cancer specific mortality in papillary thyroid cancer patients with different N category (SEER database years of 2004–2016).

**PTC**	**Mortality**	**Unadjusted**		**Adjusted^a^**	
	***n/N*** **(%)**	**HR (95%CI)**	***P***	**HR (95%CI)**	***P***
All N	234/561 (41.71)				
≤ 2 cm	51/177 (28.81)	Ref.
>2 cm	183/384 (47.66)	2.04 (1.50–2.79)	<0.001	1.99 (1.45–2.74)	<0.001
≤ 3 cm	91/288 (31.60)	Ref.
>3 cm	143/273 (52.38)	2.15 (1.65–2.80)	<0.001	2.22 (1.69–2.09)	<0.001
N0	68/203 (33.50)	
≤ 2 cm	22/84 (26.19)	Ref.
>2 cm	46/119 (38.66)	1.53 (0.92–2.55)	0.100	1.68 (1.00–2.82)	0.051
≤ 3 cm	32/121 (26.45)	Ref.
>3 cm	36/82 (43.90)	1.74 (1.08–2.80)	0.023	2.02 (1.24–3.31)	0.005
N1	151/327 (46.18)	
≤ 2 cm	24/81 (29.63)	Ref.
>2 cm	127/246 (51.63)	2.34 (1.51–3.63)	<0.001	2.08 (1.33–3.26)	0.001
≤ 3 cm	52/148 (35.14)	Ref.
>3 cm	99/179 (55.31)	2.19 (1.56–3.08)	<0.001	2.08 (1.47–2.94)	<0.001

*PTC, papillary thyroid cancer*.

a *Adjusted for age, gender, race, and radiation treatment*.

**Table 5:** The threshold of tumor size concerning thyroid cancer specific mortality in papillary thyroid cancer patients with or without extrathyroidal extension (SEER database years of 2004–2016).

**PTC^a^**	**Mortality**	**Unadjusted**		**Adjusted^b^**	
	***n/N*** **(%)**	**HR (95%CI)**	***P***	**HR (95%CI)**	***P***
ETE	165/324 (50.93)				
≤ 2 cm	19/64 (29.69)	Ref.
>2 cm	146/260 (56.15)	2.57 (1.59–4.16)	<0.001	2.35 (1.44–3.83)	0.001
≤ 3 cm	52/126 (41.27)	Ref.
>3 cm	113/198 (57.07)	1.85 (1.33–2.58)	<0.001	1.79 (1.28–2.51)	0.001
Non–ETE	63/221 (28.51)				
≤ 2 cm	31/109 (28.44)	Ref.
>2 cm	32/112 (28.57)	1.017 (0.62–1.67)	0.948	0.98 (0.59–1.63)	0.948
≤ 3 cm	37/153 (24.18)	Ref.
>3 cm	26/68 (38.24)	1.84 (1.12–3.05)	0.017	1.90 (1.12–3.22)	0.017

*PTC, papillary thyroid cancer; ETE, extrathyroidal-extension*.

a *16 PTC patients without information of extrathyroidal extension were excluded*.

b *Adjusted for age, gender, race, radiation treatment, and lymph node metastasis*.

### Kaplan-Meier Analyses of TC-Specific Survival of TC Patients in Stage IVB

**Figure 1:**
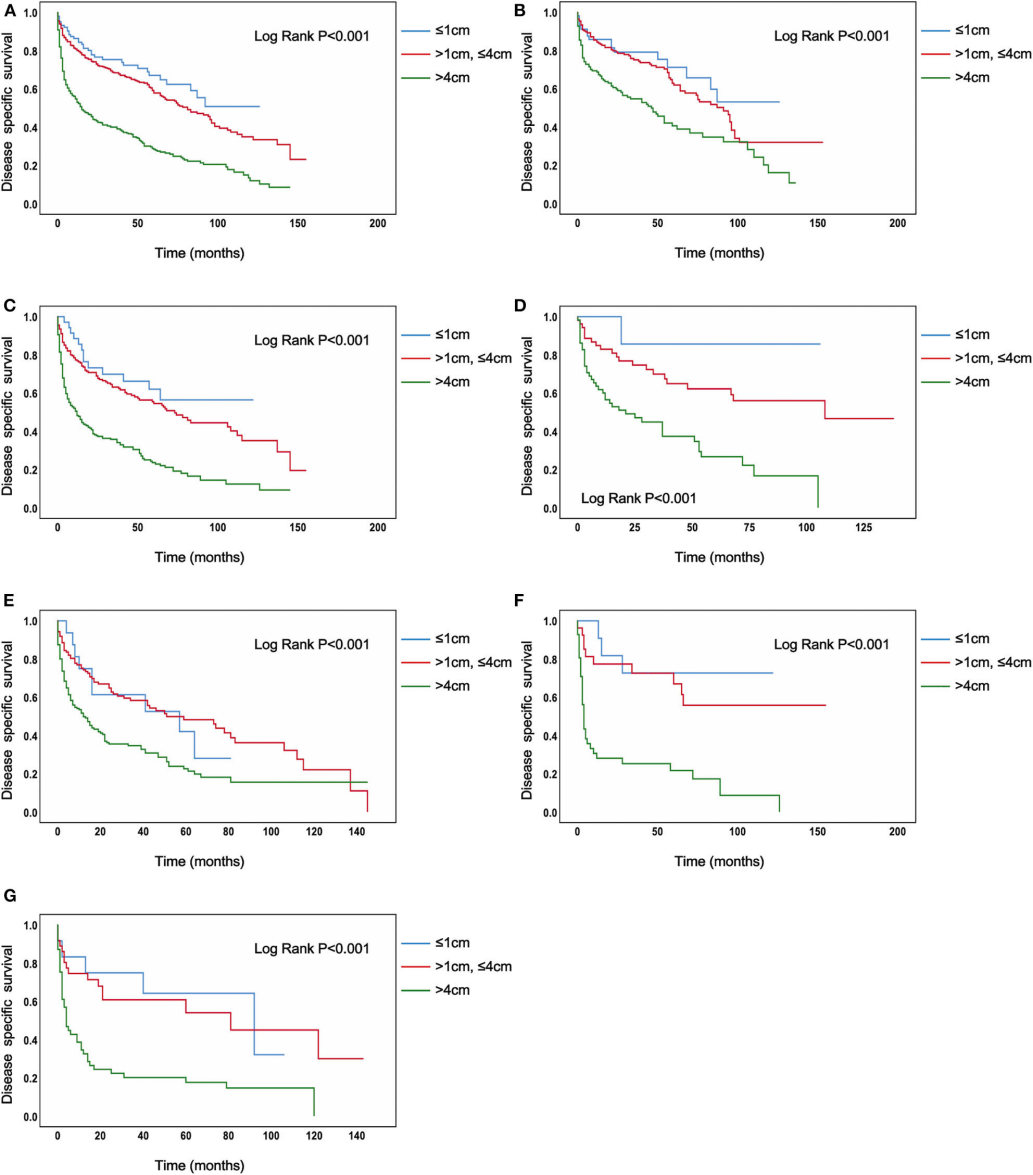
Disease specific survival of thyroid cancer patients with IVB stage stratified by tumor size using Kaplan–Meier analysis and log-rank tests. **(A)** All thyroid cancer patients. **(B)** Thyroid cancer patients with N0 category. **(C)** Thyroid cancer patients with N1 category. **(D)** Thyroid cancer patients with N1A category. **(E)** Thyroid cancer patients with N1B category. **(F)** Thyroid cancer patients with N1NOS category. **(G)** Thyroid cancer patients with Nx category. (All Log Rank *P* < 0.001).

**Figure 2:**
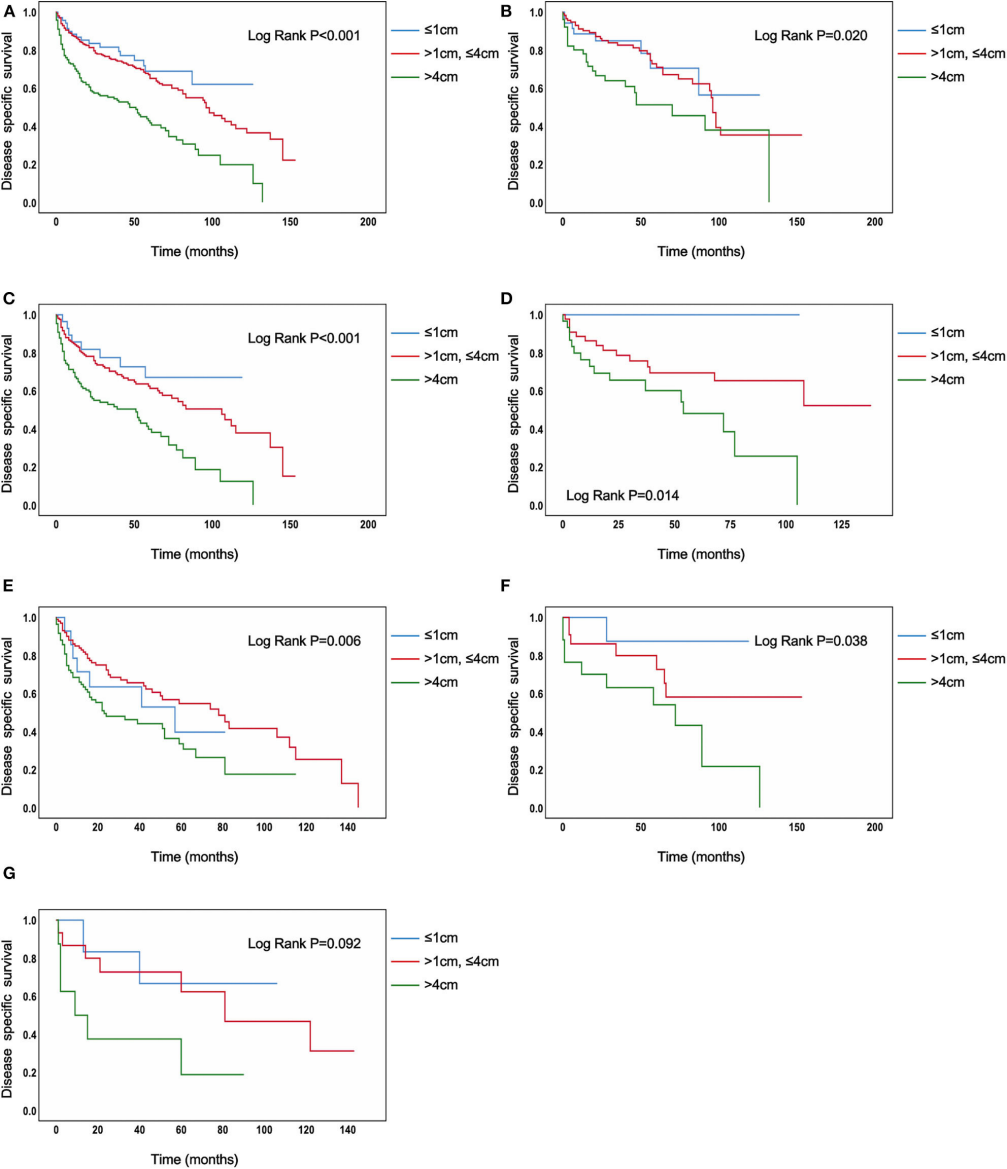
Disease specific survival of papillary thyroid cancer patients with IVB stage stratified by tumor size using Kaplan–Meier analysis and log-rank tests. **(A)** All papillary thyroid cancer patients. **(B)** Papillary thyroid cancer patients with N0 category. **(C)** Papillary thyroid cancer patients with N1 category. **(D)** Papillary thyroid cancer patients with N1A category. **(E)** Papillary thyroid cancer patients with N1B category. **(F)** Papillary thyroid cancer patients with N1NOS category. **(G)** Papillary thyroid cancer patients with Nx category [**(B–F)** Log Rank *P* < 0.050, **(G)** Log Rank *P* = 0.092].

In the analysis of TC patients in stage IVB, the increased tumor size was associated with a statistically significant decrease in the TC-specific survival curve (Log-rank *P* < 0.001) (Figure 1A). Similar trends were also observed in TC patients with N0 category (*P* < 0.001) (Figure 1B), in TC patients with N1 category (*P* < 0.001) (Figure 1C), in TC patients with N1a category (*P* < 0.001) (Figure 1D), in TC patients with N1b category (*P* < 0.001) (Figure 1E), in TC patients with N1NOS category (*P* < 0.001) (Figure 1F), and in TC patients with Nx category (*P* < 0.001) (Figure 1G). The curves of tumors >4 cm group had a sharp decrease and the curves of tumors >1 cm but ≤ 4 cm group had a moderate decrease, while tumors ≤ 1 cm group had the best survival. Similar results could be obtained in PTC patients (*P* < 0.001) (Figure 2A), in PTC with N0 category (*P* = 0.020) (Figure 2B), in PTC with N1 category (*P* < 0.001) (Figure 2C), in PTC with N1a category (*P* = 0.014) (Figure 1D), in PTC with N1b category (*P* = 0.006) (Figure 2E), and in PTC with N1NOS category (*P* = 0.038) (Figure 2F), except that in PTC with Nx category (*P* = 0.092) (Figure 2G). In FTC patients, tumors >4 cm had the worst survival, while the tumors ≤ 1 cm group had the best survival (*P* = 0.030) (Supplementary Figure 1A). Tumor size had no significant effect on ATC-specific survival (*P* = 0.333) (Supplementary Figure 2).

## Discussion

IVB stage tumors were categorized as high risk tumors in the latest American Thyroid Association guidelines (22) without further stratification by tumor size. The present study aimed to investigate the prognostic value on cancer-specific mortality of tumor size in TC patients in stage IVB. We illustrated a higher TC-specific mortality with increased tumor size and demonstrated the possibility of further stratification of the AJCC IVB stage. Tumor size clearly divided the risk stratification of PTC-specific mortality with a threshold of 3 cm irrespective of N category. However, the N category should be taken into consideration when using a threshold of 2 cm.

The results of our study for all TC cases were consistent with previous studies (19, 23, 24) that reported the adverse impact of tumor size on the prognosis of TCs. In all patients, tumor size >4 cm was the most robust risk factor of TC-specific mortality without considering the impact of the N category. Similar results could be obtained in patients in the N1 category. However, for patients in the N0 category, a tumor size of >4 cm became an independent risk factor only after adjusting for age, gender, race, and radiation treatment. In context to this, Tran et al. (25) reported in PTC patients over 55 years, tumor size over 2 cm was associated with a higher risk of recurrence compared with those under 2 cm. However, in terms of prognosis, we focused on TC-specific mortality and considered a tumor size >4 cm as an independent risk factor, while Tran et al. focused more on the aspect of recurrence-free survival and concluded a single size threshold of 2 cm. Furthermore, Nguyen et al. (26) illustrated that all-cause mortality was only elevated when the tumor size was >2.5 cm. Compared with their study, the selected population of our study had a more advanced and aggressive stage, which may result in a larger tumor size cutoff for risk stratification (4 vs. 2.5 cm).

In order to determine the exact threshold, we divided the patients by tumor size into more detailed subgroups. Higher risk of PTC-specific mortality was observed in tumors >2 cm group but the predictive value of the 2 cm threshold was limited by N category and extrathyroidal extension. Strikingly, in all PTC cases, a tumor size of 3 cm was observed to be more significant than 4 cm and irrespective of N category and extrathyroidal extension, and this phenomenon could also be found in the staging system and guidelines as cut-off (22, 27). To the best of our knowledge, this is the first study in which the specific mortality risk associated with tumor size classified by 3 cm has been evaluated. The AJCC staging manual is world-renown as the golden standard of the staging system. There are many similarities in the definition of the staging groups between the 7th and 8th editions. In many surveys (18, 28), the feasibility and superiority of the latest edition have been reported despite identifying tumor size as an important impact factor for classifying stage IVB. Nevertheless, some researchers have revealed flaws in the staging system. In a study from the MD Anderson Cancer Center (29) that enrolled 2,323 DTC cases, tumor size was highlighted as an independent factor that has predictive value of disease-free survival for patients in stage M0, and researchers of another study (18) of DTC patients from the same center reported no significant differences in disease-free survival between stage III and stage IV as classified by the AJCC staging system 8th edition. For a long time, tumor size was regarded as an important factor for different stages (30, 31), but not stage IVB. We demonstrated that tumor size was a strong mortality risk factor for such patients, particularly for those with tumors over 3 cm for PTC. Our striking findings provided evidence for further stratification in stage IVB, which may lead to changes in the risk stratification and clinical treatments.

Patients in stage IVB tumors are often treated with a combination of radioiodine therapy, radiation therapy, and surgery along with active surveillance due to the progressiveness and advancement of the tumor (21, 32). All treatment recommendations from the guidelines of the American Thyroid Association (ATA) (22) were based on the staging system, which can reinforce patient care. However, IVB patients with different tumor sizes were treated equally, while their risks of cancer-specific death were quite different. This study documented 3 cm as a threshold for TC-specific mortality in stage IVB patients, which can not only provide more information but also make more aggressive treatment reasonable for these patients with advanced stages and large tumor sizes. Clinically, radiation treatment is the most common therapy for advanced TC patients. Given the poor prognosis of IVB patients with tumor size >3 cm, an increased dose of radioactive iodine within the suitable range was presumed to be necessary, but the exact dose and clinical trials with large sample sizes still requires further investigation. Many researchers have emphasized the importance of counting gene mutations (e.g., BRAF) as a key risk factor for patients with stage IVB (1, 33, 34). Xing et al. (35–37) reported that the mutation of BRAF V600E differentiated PTC into low- and high-risk groups. Recently, more comprehensive studies have come up and broadened the understanding of DTC in aspect of molecules. In addition to BRAF mutation, TERT promoter mutation is another factor that could help identify the high-risk group in advanced TC (38). Additionally, over the last decades, several new drugs and new methods of targeted therapy have been launched (12). A study conducted in France reported 75 patients with advanced radioactive iodine-refractory DTC could benefit from Lenvatinib with a median progression-free survival of 10 months (39), and this drug was recently approved by the U.S. Food and Drug Administration. More attention should be paid to targeted therapy with the better understanding of risk stratification of TC for precise treatment.

As a retrospective study, this research had its inherent limitations, such as selection bias. All data collected from the SEER database may be influenced by the small possibility of coding errors; however, it is not a huge problem because the database is standardized and highly audited (16). Also, the information on recurrence and gene mutation was not available.

In conclusion, the risk of TC-specific mortality was found to increase with tumor size in IVB patients. For PTC patients with IVB stage, our study clearly presented a threshold of 3 cm concerning the risk of TC-specific mortality irrespective of N category, which demonstrated the possibility of further stratification in IVB stage classified by the 8th edition of AJCC Staging Manual, and may justify the necessity of a more aggressive treatment for patients with tumors over 3 cm.

## Data Availability Statement

The datasets analyzed for this study can be found in the SEER database at https://seer.cancer.gov/.

## Ethics Statement

The authors are accountable for all aspects of the work in ensuring that questions related to the accuracy or integrity of any part of the work are appropriately investigated and resolved. All the data investigated in this study was obtained from the SEER database, which was publicly available, and we received permission for using the data for non-commercial use. This study was exempt by the ethics committee of Shanghai Tenth People's Hospital.

## Author Contributions

JZ, XC, BS, YH, and SQ: conception and design. All authors: acquisition, statistical analysis or interpretation of the data, drafting of the manuscript, reviewing, and approving the final version of the manuscript.

## Conflict of Interest

The authors declare that the research was conducted in the absence of any commercial or financial relationships that could be construed as a potential conflict of interest.
