# Supplementary material for: The Increased Risk of Thyroid Cancer-Specific Mortality With Tumor Size in Stage IVB Patients
Source: Front Oncol. 2020 Nov 5;10:560203. doi: 10.3389/fonc.2020.560203 (PMC7678015; doi:10.3389/fonc.2020.560203)
Supplement: Supplementary file 1 [file Data_Sheet_1.docx]

Supplementary Material

# Supplementary Table 1. The general information of tumor size and lymph node category in thyroid cancer and its common variants (SEER database years of 2004–2016).

| Characteristics |  | TC | | PTC | | FTC | | ATC | | Other | |
| --- | --- | --- | --- | --- | --- | --- | --- | --- | --- | --- | --- |
|  |  | Number | % | Number | % | Number | % | Number | % | Number | % |
| Overall | N | 1,345 | | 763 | | 259 | | 115 | | 208 | |
|  | ≤1cm | 149 | 11.08 | 101 | 13.24 | 31 | 11.97 | 3 | 2.61 | 14 | 6.73 |
|  | >1cm but ≤4cm | 575 | 42.75 | 405 | 53.08 | 91 | 35.14 | 18 | 15.65 | 61 | 29.33 |
|  | >4cm | 621 | 46.17 | 257 | 33.68 | 137 | 52.90 | 94 | 81.74 | 133 | 63.94 |
| N0 | N | 535 | | 282 | | 171 | | 28 | | 54 | |
|  | ≤1cm | 82 | 15.33 | 51 | 18.09 | 24 | 14.04 | 2 | 7.14 | 5 | 9.26 |
|  | >1cm but ≤4cm | 255 | 47.66 | 156 | 55.32 | 69 | 40.35 | 6 | 21.43 | 24 | 44.44 |
|  | >4cm | 198 | 37.01 | 75 | 26.60 | 78 | 45.61 | 20 | 71.43 | 25 | 46.30 |
| N1 | N | 670 | | 436 | | 53 | | 64 | | 117 | |
|  | ≤1cm | 51 | 7.61 | 43 | 9.86 | 2 | 3.77 | 1 | 1.56 | 5 | 4.27 |
|  | >1cm but ≤4cm | 273 | 40.74 | 227 | 52.06 | 10 | 18.87 | 11 | 17.19 | 25 | 21.37 |
|  | >4cm | 346 | 51.64 | 166 | 38.07 | 41 | 77.36 | 52 | 81.25 | 87 | 74.36 |
| Nx | N | 140 | | 45 | | 35 | | 23 | | 37 | |
|  | ≤1cm | 16 | 11.43 | 7 | 15.56 | 5 | 14.29 | 0 | 0.00 | 4 | 10.81 |
|  | >1cm but ≤4cm | 47 | 33.57 | 22 | 48.89 | 12 | 34.29 | 1 | 4.35 | 12 | 32.43 |
|  | >4cm | 77 | 55.00 | 16 | 35.56 | 18 | 51.43 | 22 | 95.65 | 21 | 56.76 |

**Footnote** PTC papillary thyroid cancer, FTC follicular thyroid cancer; ATC anaplastic thyroid cancer, Other other variants of thyroid cancer. According to the American Joint Committee on Cancer (AJCC) Staging Manual 6^th^ Edition, lymph node category was classified into 5 groups as follows: no metastatic nodes (N0); nodes not assessed at surgery (Nx); metastases to level VI [pretracheal, paratracheal, and prelaryngeal/Delphian lymph nodes] (N1a); metastasis to unilateral, bilateral, or contralateral cervical or superior mediastinal mode metastases (N1b), and; metastasis to regional lymph nodes but not otherwise specified (N1NOS). N1stage includes N1a category, N1b category, and N1NOS category.

# Supplementary Table 2. The threshold of tumor size concerning thyroid cancer specific mortality in follicular thyroid cancer patients (SEER database years of 2004–2016).

| FTC | Mortality | Unadjusted |  | Adjusted ^a^ |  |
| --- | --- | --- | --- | --- | --- |
|  | n/N (%) | HR (95%CI) | P | HR (95%CI) | P |
| All N | 91/207(43.96) |  |  |  |  |
| ≤1cm | 6/23 (26.09) | Ref. | | | |
| >1cm but ≤4cm | 26/72 (36.11) | 1.44 (0.59-3.51) | 1.440 | 1.42 (0.55-3.70) | 0.470 |
| >4cm | 59/112 (52.68) | 2.31 (0.99-5.36) | 0.052 | 2.06 (0.87-4.86) | 0.099 |
| ≤2cm | 12/42(28.57) | Ref. | | | |
| >2cm | 79/165(47.88) | 1.82(0.991-3.35) | 0.054 | 1.67(0.89-3.12) | 0.107 |
| ≤3cm | 23/75(30.67) | Ref. | | | |
| >3cm | 68/132(51.52) | 1.82(1.13-2.92) | 0.014 | 1.69(1.02-2.79) | 0.040 |
| N0 | 55/136(40.44) |  |  |  |  |
| ≤1cm | 5/17 (29.41) | Ref. | | | |
| >1cm but ≤4cm | 23/57 (40.35) | 1.37 (0.52-3.62) | 0.522 | 2.07 (0.70-6.18) | 0.191 |
| >4cm | 27/62 (43.55) | 1.30 (0.50-3.43) | 0.590 | 1.30 (0.48-3.53) | 0.605 |
| ≤2cm | 11/32(34.38) | Ref. | | | |
| >2cm | 44/104(42.31) | 1.22(0.63-2.36) | 0.563 | 1.17(0.58-2.34) | 0.662 |
| ≤3cm | 21/60(35.00) | Ref. | | | |
| >3cm | 34/76(44.74) | 1.22(0.70-2.11) | 0.484 | 1.07(0.59-1.95) | 0.825 |
| N1 | 24/41(58.54) |  | | | |
| ≤1cm | 0/2 (0.00) | - ^b^ | | | |
| >1cm but ≤4cm | 2/4 (50.00) | - ^b^ | - ^b^ | - ^b^ | - ^b^ |
| >4cm | 22/35 (62.86) | - ^b^ | - ^b^ | - ^b^ | - ^b^ |
| ≤2cm | 0/2(0.00) | - ^b^ | | | |
| >2cm | 24/39(61.54) | - ^b^ | - ^b^ | - ^b^ | - ^b^ |
| ≤3cm | 1/4(25.00) | Ref. | | | |
| >3cm | 23/37(62.16) | 4.55(0.61-34.05) | 0.140 | 4.79(0.58-39.75) | 0.146 |

**Footnote** FTC follicular thyroid cancer. 207 patients with information of FTC-specific mortality and tumor size were analyzed, including 136 FTC patients with N0 category, 41 FTC patients with N1 category and 30 patients with Nx category. ^a^ Adjusted for age, gender, race and radiation treatment. ^b^ The values of HRs and P values cannot be calculated because of no death in reference group.
