# Supplementary figures and images for: The Increased Risk of Thyroid Cancer-Specific Mortality With Tumor Size in Stage IVB Patients
Source: Front Oncol. 2020 Nov 5;10:560203. doi: 10.3389/fonc.2020.560203 (PMC7678015; doi:10.3389/fonc.2020.560203)

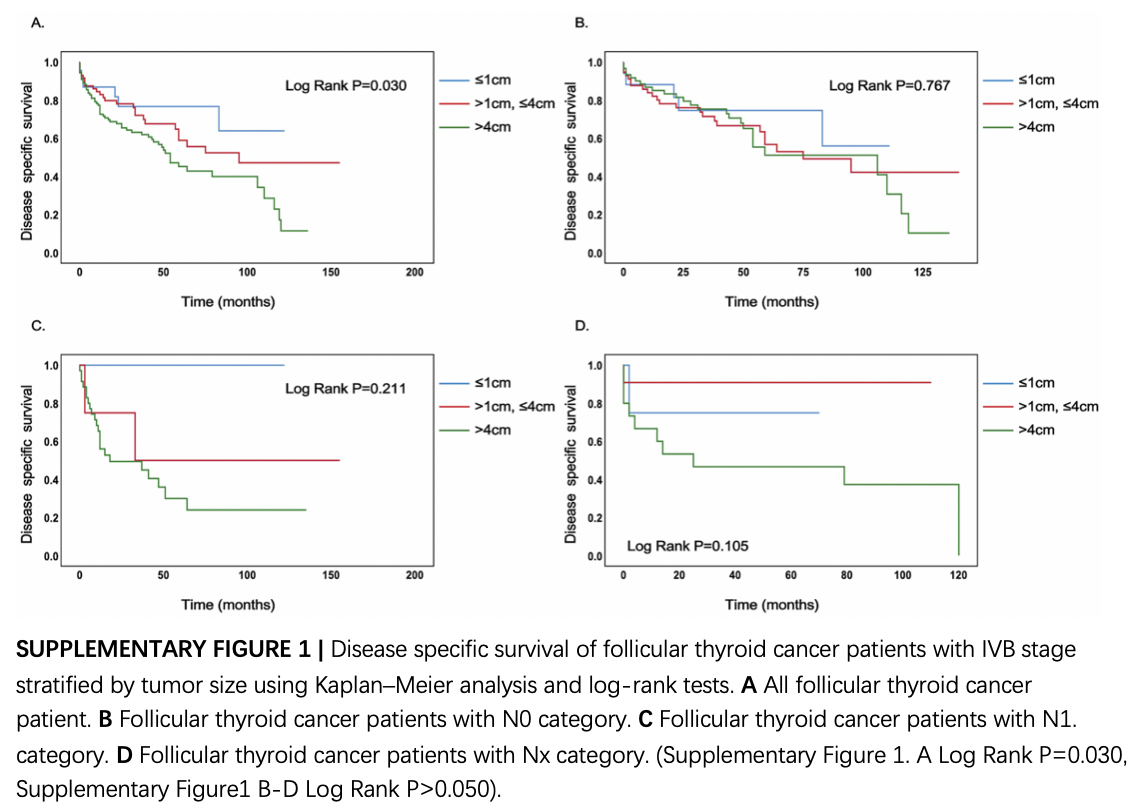

Supplement: Supplementary file 2 [file Image_1.PNG]

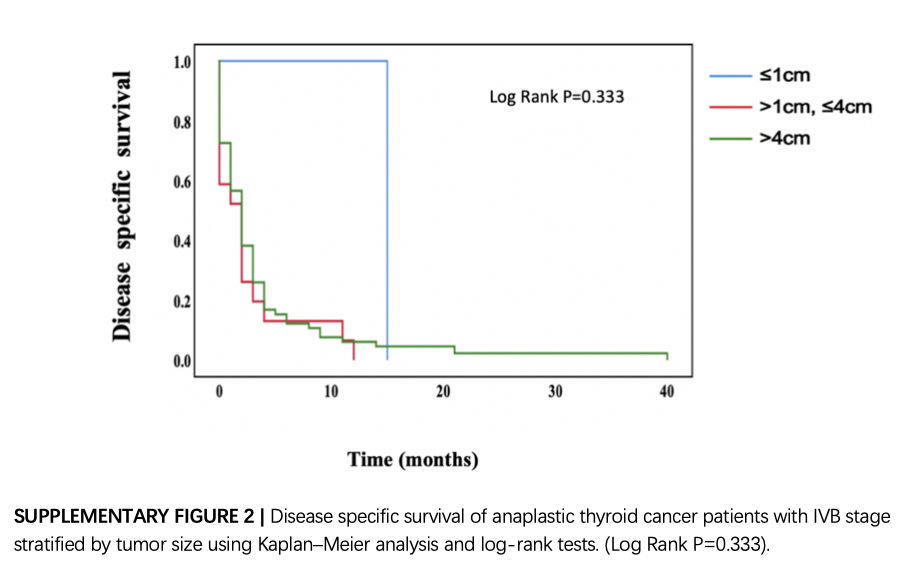

Supplement: Supplementary file 3 [file Image_2.PNG]
